# Tumour-Infiltrating Inflammatory Cells in Early Breast Cancer: An Underrated Prognostic and Predictive Factor?

**DOI:** 10.3390/ijms21218238

**Published:** 2020-11-03

**Authors:** Sören Schnellhardt, Ramona Erber, Maike Büttner-Herold, Marie-Charlotte Rosahl, Oliver J. Ott, Vratislav Strnad, Matthias W. Beckmann, Lillian King, Arndt Hartmann, Rainer Fietkau, Luitpold Distel

**Affiliations:** 1 Department of Radiation Oncology, Universitätsklinikum Erlangen, Friedrich-Alexander-Universität Erlangen-Nürnberg, D-91054 Erlangen, Germany; soeren.schnellhardt@fau.de (S.S.); marie.rosahl@gmail.com (M.-C.R.); Oliver.ott@uk-erlangen.de (O.J.O.); vratislav.strnad@uk-erlangen.de (V.S.); rainer.fietkau@uk-erlangen.de (R.F.); 2 Institute of Pathology, Universitätsklinikum Erlangen, Comprehensive Cancer Center Erlangen-EMN, Friedrich-Alexander-Universität Erlangen-Nürnberg, D-91054 Erlangen, Germany; Ramona.erber@uk-erlangen.de (R.E.); Arndt.Hartmann@uk-erlangen.de (A.H.); 3 Department of Nephropathology, Institute of Pathology, Universitätsklinikum Erlangen, Friedrich-Alexander-Universität Erlangen-Nürnberg, D-91054 Erlangen, Germany; Maike.Buettner-Herold@uk-erlangen.de; 4 Department of Gynecology and Obstetrics, Universitätsklinikum Erlangen, Comprehensive Cancer Center Erlangen-EMN, Friedrich-Alexander-Universität Erlangen-Nürnberg, D-91054 Erlangen, Germany; fk-direktion@uk-erlangen.de; 5 Intensive Care Unit, QEII Jubilee Hospital, Brisbane, Queensland 4108, Australia; Lillian.jy.king@gmail.com

**Keywords:** breast cancer, CD4, CD45RO, CD20, CD1a, hormone receptor positive, accelerated partial breast irradiation, TIL, immunoscore, prognosis

## Abstract

The role of tumour-infiltrating inflammatory cells (TIICs) in the disease progression of hormone-receptor-positive breast cancer (HR+ BC) is largely unclear since it is generally regarded as the least immunogenic BC subtype. This study investigated the prognostic significance of CD1a+ dendritic cells, CD20+ B cells, CD45RO+ memory T cells and CD4+ T-helper cells in HR+ BC. One hundred and forty-six patients were treated for early stage, distant-metastases-free HR+ BC in an accelerated partial breast irradiation (APBI) phase II trial. Immunohistochemistry was used to double-stain two adjoining sets of tissue microarrays from pre-RT (radiotherapy) tumour resection samples for CD1a/CD20 and CD45RO/CD4. Cell densities of CD1a+, CD20+, CD45RO+ and CD4+ TIICs in the stromal and intraepithelial compartment were registered semiautomatically. High densities of CD20+ and CD4+ TIICs were strongly associated with reduced disease-free survival (DFS), while high stromal CD45RO+ TIIC densities were indicators of subsequent successful treatment. An immunoscore based on CD20+ and CD45RO+ TIIC densities identified three different risk groups (*p* < 0.001). Thus, contrary to current assumptions, intratumoural immune cell composition might be an important prognostic indicator and a possible contributing factor in the outcome of HR+ BC and should be the subject of further research. Specifically, B-cell infiltration entailed an increased relapse rate and could play an important role in disease progression.

## 1. Introduction

Understanding the role of tumour-infiltrating inflammatory cells (TIICs) in the development and progression of malignant tumours has become a key aspect of cancer research and is essential for the development of novel therapies and prognostic tools [1]. The progress achieved towards this goal is variable for different types of cancers. In the treatment of malignant melanoma, immunotherapies are already part of everyday clinical practice [2]. In colorectal carcinoma, there is an initiative to expand the TNM classification through the addition of a prognostic immunoscore [3]. However, in breast cancer (BC), the role of the immune system remains unclear and continuous efforts are still being made to understand the prognostic influence of several types of immune cells [4]. The different molecular breast cancer subtypes make this endeavour even more challenging, as they seem to vary greatly in their immunogenicity. Triple-negative BC (TNBC) and human-epidermal-growth-factor-2-positive/hormone-receptor-negative (Her2+/HR-) tumours, on the one hand, are considered immunogenic [5]. A generally unfavourable outcome, high densities of TIICs and numerous reports of their association with improved prognosis have made this group an obvious and promising target for immunological research [6,7,8,9].

Hormone-receptor-positive breast cancer (HR+ BC) of the luminal subtype, on the other hand, is studied considerably less frequently in this respect, despite the fact that it is the most prevalent type of breast cancer [10]. This is partly because of the overall more favourable outcome in this group, but also due to an apparent lack of immunogenicity resulting in relatively low TIIC densities, which seem to be without prognostic significance [11,12]. Only isolated results have suggested that TIICs might also play a part in disease progression of HR+ tumours [13].

In order to gain a better understanding of the complex role of the immune system in this subgroup, it has been proposed that an analysis of individual types of immune cells in homogeneous patient cohorts with minimal interfering factors might be necessary [14]. Following this suggestion, we could already report a surprisingly strong prognostic relevance of tumour-associated macrophages (TAMs) in luminal breast cancer [15]. To expand upon these findings, this study further analysed this cohort for a diverse spectrum of immune cells that are still relatively unexplored in this context. The results indicate that TIIC composition might have a critical influence on the outcome of HR+ BC, thus challenging previous assumptions of a subordinate role of the immune system in this subgroup of breast cancer.

## 2. Results

**Figure 1:**
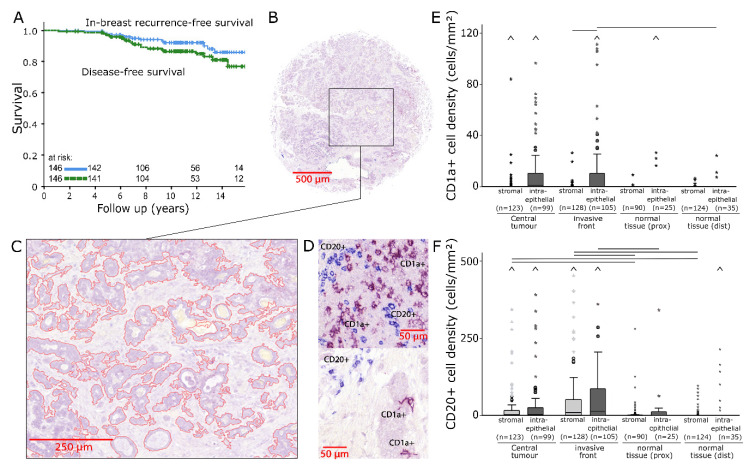
(**A**) In-breast recurrence-free survival and disease-free survival of the studied patient cohort analysed with the Kaplan–Meier method and log-rank test. (**B**) Image of a representative breast cancer tissue microarray core double-stained for CD1a/CD20 (200× magnification). (**C**) Intraepithelial tumour compartment marked with image analysis software. (**D**) Representative examples of CD1a+ (violet) and CD20+ (blue) tumour-infiltrating inflammatory cells (TIICs). (**E**,**F**) Stromal and intraepithelial cell density distributions of CD1a+ (**E**) and CD20+ TIICs (**F**) in samples from four different locations. The central line represents median values, while the box is indicative of the interquartile range (IQR). Whiskers represent 1.5× IQR or minimum/maximum. Outliers are indicated by points (up to 3× IQR) or asterisks (>3 IQR). Outliers not visible in this diagram are depicted as a caret. Black bars signify *p* < 0.05 in Student’s *t* test.

One hundred and forty-six patients were treated for early stage HR+ BC (Table 1) and the resulting 12-year disease-free survival (DFS) was 85% (Figure 1A). Two adjoining sets of tissue microarray (TMA) slides produced from pre-RT (radiotherapy) tissue samples from four locations, namely, central tumour (CT), invasive front (IF), normal tissue taken from tumour proximity (prox) and normal tissue that was sampled distant from the tumour (dist), were analysed for each patient.

### 2.1. CD1a/CD20 Immunostaining

For each location, a double-immunostaining for CD1a/CD20 was applied to one of the two TMAs (Figure 1B). The area of the intraepithelial and stromal compartments (Figure 1C) and numbers of stained cells were quantified in each TMA core (Figure 1D). Then, in both compartments, cell densities of CD1a+ and CD20+ TIICs were calculated separately in four independent sample locations.

CD1a+ dendritic cells occurred in relatively low densities and were predominantly counted in tumour epithelium (Figure 1E). CD1a+ cell densities were not associated with DFS.

**Figure 2:**
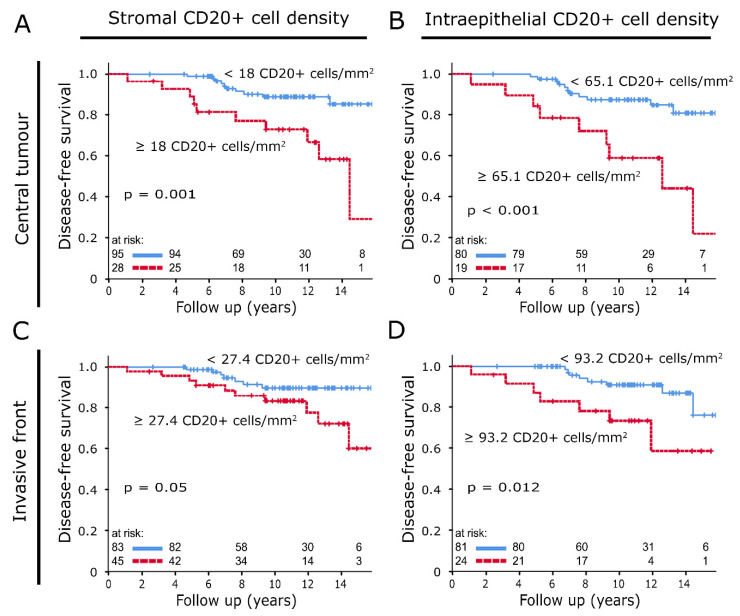
(**A**,**B**) Disease-free survival analysed with the Kaplan–Meier method and log-rank test according to CD20+ tumour-infiltrating inflammatory cell (TIIC) densities in the stromal (**A**) and intraepithelial compartment (**B**) of central tumour samples. (**C**,**D**) Disease-free survival analysed with the Kaplan–Meier method and log-rank test according to CD20+ TIIC densities in the stromal (**C**) and intraepithelial compartment (**D**) of invasive front samples.

CD20+ cell densities were characterised by a diffuse infiltration of tumour epithelium and stroma. The presence of occasional cell clusters resulted in high variance (Figure 1F; CT stromal: median: 2.7 cells/mm^2^, standard deviation (SD): 973 cells/mm^2^; IF stromal: median: 9.0 cells/mm^2^, SD: 697 cells/mm^2^). These cell clusters were not found in normal tissue, where CD20+ cell density distributions, especially in the stromal compartment, were significantly lower (*p* < 0.05). In both compartments of CT and IF, correlations were found between high densities of CD20+ B lymphocytes and reduced DFS. In CT (stromal: Figure 2A: *p* = 0.001; intraepithelial: Figure 2B, *p* < 0.001) and in the intraepithelial compartment of IF, these correlations were distinct (stromal: Figure 2C, *p* = 0.05; intraepithelial: Figure 2D, *p* = 0.012).

### 2.2. CD45RO/CD4 Immunostaining

**Figure 3:**
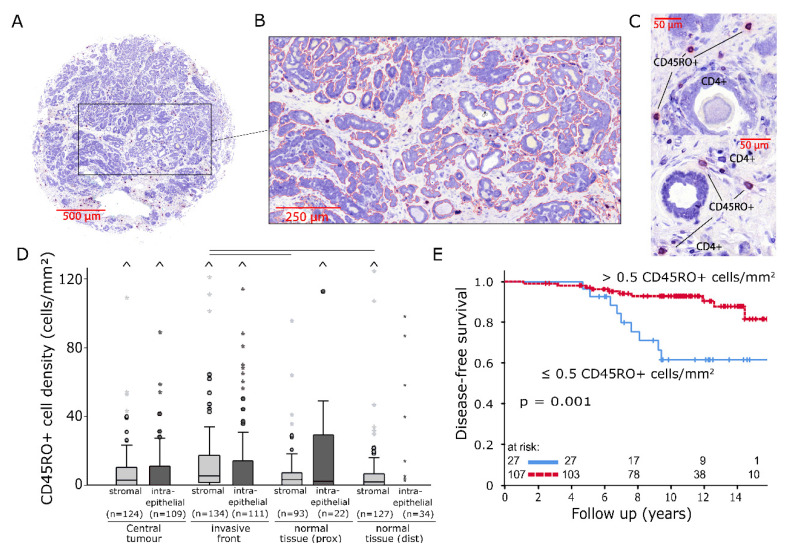
(**A**) Image of a representative breast cancer tissue microarray core double-stained for CD45RO/CD4 (200× magnification). (**B**) Intraepithelial tumour compartment marked with image analysis software. (**C**) Representative examples of CD45RO+ (violet) and CD4+ (blue) tumour-infiltrating inflammatory cells (TIICs). (**D**) Stromal and intraepithelial cell density distributions of CD45RO+ TIICs in samples from four different locations. The central line represents median values, while the box is indicative of the interquartile range (IQR). Whiskers represent 1.5× IQR or minimum/maximum. Outliers are indicated by points (up to 3× IQR) or asterisks (>3 IQR). Outliers not visible in this diagram are depicted as a caret. Black bars signify *p* < 0.05 in Student’s *t* test. (E) Disease-free survival analysed with the Kaplan–Meier method and log-rank test according to CD45RO+ TIIC densities in the stromal compartment of invasive front samples.

Another set of TMAs was stained for CD45RO+ and CD4+ cells (Figure 3A). Again, cell densities were calculated by determining the area of the stromal and intraepithelial compartments (Figure 3B) as well as the number of stained cells (Figure 3C). CD45RO+ TIICs were overall characterised by relatively low cell densities and poor epithelial infiltration (Figure 3D; CT stromal: median: 2.8 cells/mm^2^, SD: 136 cells/mm^2^; IF stromal: 5.6 cells/mm^2^, SD: 34 cells/mm^2^). Noticeable accumulations of CD45RO+ cells in comparison to normal tissue were observed in the stromal compartment of IF (*p* < 0.05). A lack of CD45RO+ memory T cells in the stromal compartment of IF was associated with a decline in DFS (Figure 3E, *p* = 0.001).

**Figure 4:**
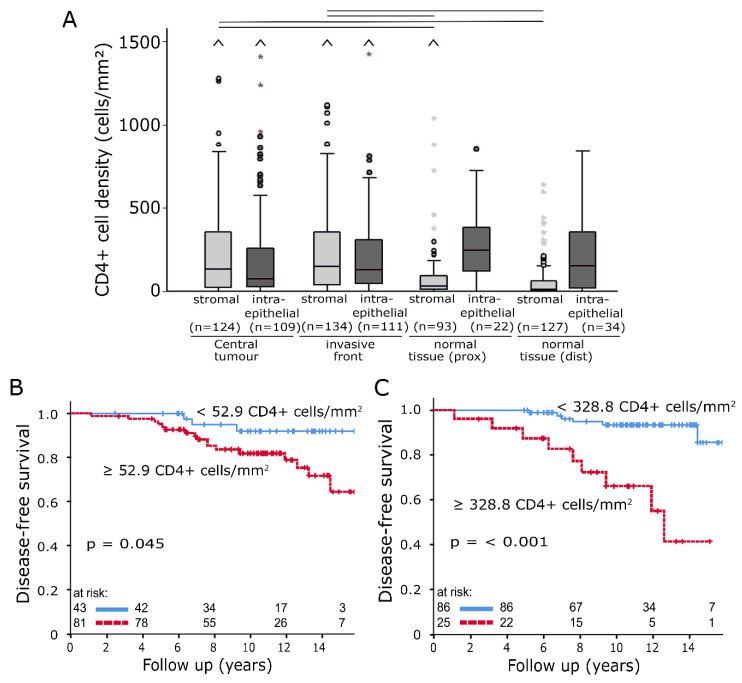
(**A**) Stromal and intraepithelial cell density distributions of CD4+ tumour-infiltrating inflammatory cells (TIICs) in samples from four different locations. The central line represents median values, while the box is indicative of the interquartile range (IQR). Whiskers represent 1.5× IQR or minimum/maximum. Outliers are indicated by points (up to 3× IQR) or asterisks (>3 IQR). Outliers not visible in this diagram are depicted as a caret. Black bars signify *p* < 0.05 in Student’s *t* test. (**B**,**C**) Disease-free survival analysed with the Kaplan–Meier method and log-rank test according to CD4+ TIIC densities in the intraepithelial compartment of invasive front samples (**B**) and in the stromal compartment of central tumour samples (**C**).

CD4+ T-helper cells were the most frequently registered TIICs in this study (Figure 4A), with stromal median values of 133.8 cells/mm^2^ (CT, standard deviation (SD): 422 cells/mm^2^) and 147.5 cells/mm^2^ (IF, SD: 322 cells/mm^2^). Tumour samples had a significantly higher level of stromal CD4+ TIIC densities than normal tissue (*p* < 0.05). High densities of CD4+ TIICs were negatively associated with DFS. There were clear correlations in the intraepithelial compartment at IF (Figure 4B, *p* < 0.001) as well as in the stromal compartment of CT (Figure 4C, *p* = 0.045).

No correlation was observed between stromal CD4+ and CD20+ TIIC densities in normal breast tissue samples. In tumour samples, a positive monotonic correlation could be observed between these cell types (Table 2).

The lobular breast cancer subtype was associated with increased epithelial CD45RO+ TIIC infiltration at IF (Table 3). No correlations were found between clinical characteristics and CD1a+, CD20+ or CD4+ TIIC densities.

Multivariate Cox regression analysis revealed stromal CD20+ and CD45RO+ TIIC densities and the proliferation index Ki67 to be independent risk factors for DFS (Table 4).

### 2.3. Immunoscore

**Figure 5:**
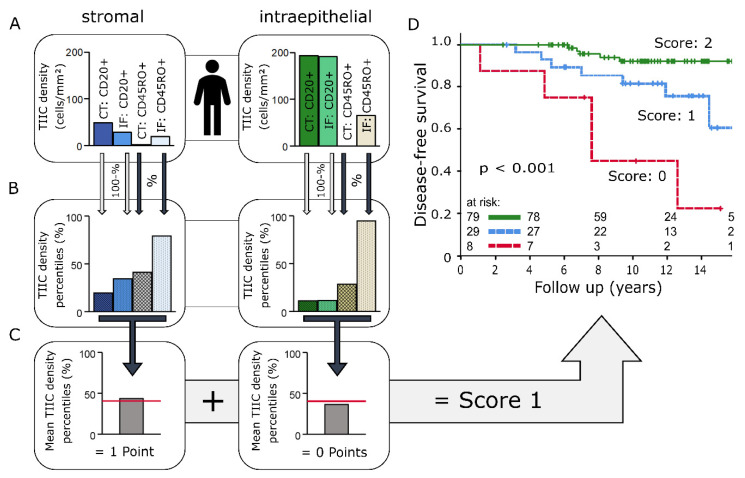
(**A**–**C**): Immunoscore model applied to results of one patient: (**A**) Observed stromal and intraepithelial CD20+ and CD45RO+ tumour-infiltrating inflammatory cell (TIIC) densities in central tumour (CT) and invasive front (IF) samples. (**B**) CD45RO+ TIIC densities ranked as percentiles within the whole cohort. CD20+ TIIC density percentiles were subtracted from 100 due to the negative prognostic effect of CD20+ TIICs. (**C**) Mean TIIC percentiles of the stromal and intraepithelial compartment. Mean percentiles above the threshold (red line) resulted in one immunoscore point per compartment. (**D**) Disease-free survival analysed with the Kaplan–Meier method and log-rank test according to immunoscore results based on stromal and intraepithelial CD20+ and CD45RO+ TIIC densities.

A prognostic immunoscore utilising CD20+ and CD45RO+ cell densities was designed based on the methodology used in the immunoscore for colorectal carcinoma [3]. CD20+ and CD45RO+ TIIC densities from CT and IF were ranked as percentiles (Figure 5A,B), and the arithmetic mean M was calculated separately for the stromal and intraepithelial compartments. To account for the opposite influences of B lymphocytes and memory T cells on DFS, CD20+ TIIC density percentiles were subtracted from 100:

$$M=\frac{\left(CD45RO^{+}TIIC\%CT+CD45RO^{+}TIIC\%IF+\left(100\;-\;CD20^{+}TIIC\%CT\right)+\left(100-CD20^{+}\;TIIC\%IF\right)\right)}{4}$$

Optimal cut-off points for mean values with regard to DFS were then calculated through receiver operating characteristic (ROC) analysis (stromal: 40.6; intraepithelial: 40.1). Samples with average percentiles above the threshold received one immunoscore point per compartment (Figure 5C). Samples that only consisted of stromal tissue automatically received one point for the intraepithelial compartment.

Patients with an immunoscore of 0 points were at a very high risk of relapse (*p* < 0.001, Figure 5D).

## 3. Discussion

In recent years, numerous studies have investigated the clinical significance of TIICs in breast cancer. Consistently, these studies found that densities of TIICs in HR+ breast cancer were relatively low and of little prognostic relevance: A meta-analysis by Mao et al. consisting of 22,964 patients concluded that high densities of TIICs in TNBC and Her2+/HR- BC served as a prognostic marker for improved DFS, whilst the HR+ subgroup remained without significant results [6]. A review by Solinas et al. reported that the majority of studies concerning the HR+ subtype concluded that TIIC levels did not carry prognostic information [12]. Only one large study which analysed 1366 luminal tumours found significant results for neoadjuvant treatment in this subgroup. However, they observed a negative association between overall survival and TIIC levels, which is contrary to observations made in more aggressive BC subtypes and thus suggests that different mechanisms are involved [13]. Because of this general lack of evidence and due to the differences in disease outcome, the more aggressive and more immunogenic subtypes TNBC and Her2+/HR- BC have become the main focus of immunological research. Meanwhile, HR+ BC has been relegated to the background—a possibly unwarranted development considering the findings of our study.

Research that addressed the differences in immunogenicity between BC subtypes concluded that immune interactions in HR+ BC may be more complex and of a different nature than in other subtypes [12,13]. Oestrogen-receptor-negative (ER-) tumours were found to carry high mutational loads and might therefore express large quantities of neoantigens, which could explain the strong immune response in this group. Mutational loads in ER+ BC appear to be significantly lower in comparison [16]. Instead, there is a greater diversity of driver alterations, which could result in more diverse tumour-derived mechanisms of immunosuppression [17]. Another possibly important factor influencing the immune cell composition in HR+ BC is oestrogen-related immunomodulation. Oestrogen has been reported to have an immunosuppressive effect in BC through the stimulation of CCL5 release in the tumour microenvironment [18,19]. In addition to oestrogen, different antihormonal therapies also seem to interact with the immune system [20]. Finally, it is also unclear whether other at-this-point-unknown subgroups within the group of HR+ tumours might have an influence on TIIC composition.

To account for this complex situation, it has been proposed to perform a qualitative assessment of individual immune cell types for their clinical relevance rather than a quantitative evaluation of the general TIIC infiltration. Furthermore, this should be done in a homogeneous cohort of patients to minimise possible known and unknown confounding factors, [14]. Known confounding factors include differences in menopausal status, tumour size and spread as well as the type of treatment. This study examined a subgroup of 146 patients who were treated as part of a phase II accelerated partial breast irradiation (APBI) trial at the Universitätsklinikum Erlangen [21,22]. The strict inclusion and treatment criteria of the trial allowed us to compare the long-term disease-free survival (DFS) of a group of mostly postmenopausal women suffering from early stage luminal breast cancer, which also expressed a relatively homogeneous set of clinicopathological characteristics, with densities of the following immune cells: CD1a+ immature myeloid dendritic cells, CD45RO+ memory T cells, CD20+ B cells and CD4+ T-helper cells.

### 3.1. CD1a+ Dendritic Cells

CD1a is a marker for immature dendritic cells [23]. In this study, CD1a+ cells were registered almost exclusively in close contact with tumour epithelium. No prognostic relevance of immature dendritic cells was observed. These findings are consistent with reports from previous studies of breast cancer cohorts with mixed molecular subtypes [24,25]. A hypothesis which could explain these observations is that the maturation and differentiation of immature dendritic cells might be suppressed in tumour proximity, rendering them nonfunctional. This is supported by reports of the exclusive presence of mature dendritic cells in peritumoural areas and their association with improved survival [26,27].

### 3.2. CD45RO+ Memory T Cells

CD45RO, an isoform of the CD45 leucocyte common antigen, is commonly used as a marker to identify central memory T cells and effector memory T cells [28,29]. The presence of memory T cells in the tumour microenvironment has been described as a positive prognostic factor in multiple forms of cancer, including colorectal carcinoma, hepatocellular carcinoma and gastric cancer [30,31,32,33]. Improved DFS associated with high densities of CD45RO+ TIICs was also reported in mixed subtype breast cancer cohorts [34,35]. In these studies, luminal tumours were characterised by low levels of CD45RO+ TIIC infiltration. The results of our study confirm this, with memory T-cell densities being relatively low compared with other TIIC types. Interestingly, the stromal compartment at IF seemed to be a focal point for CD45RO+ cell activity. At this location, a significant accumulation of memory T cells could be observed in comparison to normal tissue and their presence correlated with improved survival. Thus, CD45RO+ memory T cells could also have a negative influence on tumour progression in HR+ breast cancer and might be an indicator for a functioning adaptive immune response.

### 3.3. CD20+ B Cells

The role of B cells in the progression of breast cancer is the subject of controversial discussion. It has been reported that CD20+ cells can have a positive, negative or no correlation with survival [36,37]. For one, this highlights the importance of assessing immune cells in breast cancer in the context of the known molecular breast cancer subtypes. In triple-negative and Her2+/HR- tumours, B cells seem to be associated with improved DFS [38,39]. Immune-response-activating measures such as IgG secretion, antigen presentation and direct cytotoxic effects are possible contributing mechanisms [40]. However, there is also ample evidence for B-cell-promoted disease progression: regulatory B cells (Bregs), among others, could play an important role in this regard by recruiting regulatory T cells (Tregs) and thus creating an immunosuppressive environment [41]. An improved antitumour response and lower densities of Tregs have been observed in B-cell-depleted mice [42]. Additionally, a recent study reported that B cells under the influence of breast cancers can promote the formation of lymph node metastases [43]. Our results indicate that these protumoural mechanisms could play a more prominent role in HR+ breast cancers. One of the main findings of this study is that high CD20+ cell densities consistently and strongly correlated with reduced DFS in both tumour locations. If Bregs, as a possible underlying mechanism, act as immunosuppressors via Treg recruitment, this would also explain the negative prognostic effect of CD4+ cells in this study. Unfortunately, there is no established single marker for Bregs that can be targeted with immunohistochemistry. There are, however, Breg subtypes which do express relevant amounts of CD20 that have been reported to contribute to disease progression in cancers [44,45].

### 3.4. CD4+ T-Helper Cells

The CD4+ subpopulation of T cells includes, among others, Th1, Th2, Th17, follicular helper (Tfh) T cells and Tregs [46]. For the various cell types, different and often opposite effects on survival have been demonstrated in breast cancer. Classically, Th1 is considered an antitumour phenotype, while Th2 promotes tumour growth [47]. Correlations between Tfh infiltrations and improved survival have been reported in HR+ BC [48]. In contrast, Tregs appear to be associated with disease progression in this subtype [49,50]. These opposing effects of different cell types make a clear prognostic classification of the heterogeneous group of CD4+ TIICs difficult. In HR- BC, improved survival was observed in studies investigating CD4+ TIICs, whereas this relationship was nonexistent or negative in HR+ groups [51,52]. Our study observed a negative association between CD4+ TIIC densities and DFS. One possible explanation for this could be a polarisation of the CD4+ cell population towards the immunosuppressive Treg and Th2 phenotypes.

Such a polarisation might be the result of multiple factors. Firstly, the strong correlations between CD20+ and CD4+ cell densities, which were present in tumour samples but not in normal breast tissue, heavily suggest a combined mechanism of action for those cell types. As mentioned above, this mechanism might be through the recruitment of immunosuppressive CD4+ subpopulations such as Tregs or Th2 by Bregs. This implies a possible key role for immunomodulatory B cells (e.g., Bregs) in the progression of HR+ BC. Another factor could be the influence of hormones on the immune system, as oestrogen not only induces the polarisation of CD4+ immune cells to the protumoural Th2 phenotype via CCL5 release but also promotes the proliferation of Tregs [18,53].

### 3.5. Immunoscore

Immunoscores can be a powerful tool for the combined use and potentiation of the prognostic information of different subtypes of immune cells. In colorectal carcinoma, the development of an immunoscore as a prognostic tool is already at an advanced stage [3]. In breast cancer, there are also initiatives to develop a similar immune-cell-based prognostic marker. The largest project is the guidelines of the international TIL working group on breast cancer. However, in the recommendations of this working group, the luminal subtype is barely mentioned due to its lack of immunogenicity [4]. We have developed an experimental scoring system for the HR+ subtype that is roughly based on the methodology used by Galon et al. in colorectal cancer. With memory T cells and CD20+ B cells, we used two markers that were identified as independent risk factors in multivariate survival analysis. The results demonstrate that different risk groups can also be determined by immune cell densities in HR+ BC. A small subgroup of tumours with on average high levels of CD20+ TIIC infiltration and low CD45RO+ cell counts in epithelium and stroma of IF and CT expressed an immune cell profile that was strongly associated with reduced DFS. Targeting and reversing a possible tumour-induced immune blockade in this subgroup could provide a critical survival benefit.

The main limitations of this study are the relatively small group size and the low number of local recurrences and distant metastases. Results should be interpreted with this in mind. The primary goal of the immunoscore shown here is not necessarily to introduce it as a clinical marker at this point, as the role of the immune system in HR+ BC is still too obscure. Instead, the distinct prognostic groups of the immunoscore combined with the remaining findings of this study strongly indicate that there might be an underrated immunological component influencing the outcome of HR+ BC. Consequently, these results should serve as encouragement for further research in the HR+ subtype.

## 4. Materials and Methods

### 4.1. Breast Cancer Patients and Clinical Data

Between 2000 and 2005, a cohort of 146 patients was treated for early stage breast cancer at the Universitätsklinikum Erlangen as part of the German–Austrian accelerated partial breast irradiation (APBI) phase II trial [54]. Table 1 displays clinical data and pathological characteristics. Inclusion criteria were histopathologically confirmed invasive breast carcinoma of any histologic type of ≤3 cm in diameter, unifocal and unicentric breast cancer growth pattern, clear resection margins of ≥2 mm in any direction, hormone sensitivity (oestrogen receptor positive (ER+)/progesterone receptor positive (PR+), ER+/PR−, ER−/PR+), histologic grade 1 or 2, no lymph vessel and no blood vessel invasion, no or microscopically involved axillary nodes (pN0/pNmi), no distant metastases and age of ≥35 years [21,22]. Breast-conserving surgery and either interstitial multicatheter pulsed-dose-rate (PDR) or high-dose-rate (HDR) brachytherapy were performed on all patients [55]. Tissue samples used for tissue microarray (TMA) construction were collected from tumour resections prior to radiotherapy. One hundred and thirty-one patients (89.7%) received hormone therapy, 10 (6.8%) were treated with chemotherapy and 8 patients (5.5%) received both treatments. For this immunological subanalysis of the German–Austrian APBI trial [21,22], breast-cancer-specific survival was 99% after a follow-up period of 12 years, and the disease-free survival rate (DFS) was 85%. In-breast recurrence-free survival was 92% in this subgroup.

Written informed consent was obtained “front door” from all patients for collection of their tissue and clinical data. The use of formalin-fixed, paraffin-embedded material from the Archive of the Institute of Pathology was approved by the Ethics Committee of the Friedrich-Alexander-University of Erlangen-Nuremberg on 24 January 2005 (21_ 19 B), waiving the need for consent to access the existing archived material.

### 4.2. TMA Construction and Immunohistochemistry

Two sets of four tissue microarrays with a diameter of 2 mm per core were processed from each of the 146 paraffin-embedded tumour resections (Figure 1B and Figure 3A). Sampling locations were the central tumour (CT), the invasive front (IF), normal tissue in tumour proximity (prox) and normal tissue distant from the tumour (dist). Following this, 2 µm tissue sections were deparaffinised in xylene and rehydrated with graded ethanol. Double-stainings for CD1a/CD20 and CD45RO/CD4 were performed using an alkaline phosphatase detection kit (POLAP-100, Zytomed Systems GmbH, Berlin, Germany) with Fast red (Sigma-Aldrich, Deisenhofen, Germany) as chromogen for CD1a and CD45RO and Fast blue (Sigma-Aldrich) as chromogen for CD4 and CD20 detection [56].

A high-throughput scanner was used to digitise the stained TMAs (Mirax Scan, Zeiss, Göttingen, Germany). Digital processing of the scans was done in Pannoramic Viewer version 1.15.4 (3D Histech, Budapest, Hungary).

The definitions released by the St Gallen International Expert Consensus on the Primary Therapy of Early Breast Cancer were used to classify intrinsic breast cancer subtypes [57]. Ki67 expression levels of ≥20% were described as an unfavourable prognostic factor in early breast cancer by Fasching et al. [58].

### 4.3. Quantification of Inflammatory Cells

The quantification of TIIC densities was conducted semiautomatically with image-processing software (Biomas, Erlangen, Germany). Inclusion criteria for TIICs were size, morphology and colour (Figure 1D and Figure 3C). The respective areas of the stromal and intraepithelial compartments were marked and registered (Figure 1C and Figure 3B). Cell densities were then calculated for each compartment separately.

TMAs were analysed from 146 patients in total. Cell density data, however, could sometimes only be registered in one compartment or in one of the two immunostainings. This resulted in fewer available datasets than the total number of patients. The majority of these discrepancies can be explained by samples which only consisted of stromal or epithelial tissue. Another factor to account for this discrepancy was loss of material during the staining process. TMAs from normal breast tissue often contained very little tissue or mostly fat, resulting in fewer datasets, especially intraepithelially, than in tumour samples.

### 4.4. Statistical Analyses

All statistical analyses were carried out in SPSS version 26 (IBM Inc., Chicago, IL, USA). Correlations were identified through Spearman’s Rho and Chi-squared test. Student’s *t* test for independent samples and Welch’s test were used to compare mean values of cell densities. Cox proportional hazards model was used to calculate hazard ratios of TIIC densities and clinicopathological characteristics. Covariates with *p* < 0.15 in univariate analysis were included in multivariate analyses. The proportional hazards assumption was verified by visual examination of the log-minus-log curves. Optimal cut-off points for TIIC density groups (low vs high) were calculated for disease-free survival (DFS) through receiver operating characteristic (ROC) curve analysis. Survival curves for DFS were plotted using the Kaplan–Meier method and compared with the log-rank test. *p*-Values < 0.05 were considered to be statistically significant.

## 5. Conclusions

In the studied cohort of patients with early HR+ breast cancer, tumour-infiltrating CD20+ B-cell, CD4+ T-helper-cell and CD45RO+ memory T-cell densities were significantly associated with DFS. Increased B-cell densities were an especially strong predictor of unfavourable DFS. CD4+ T-helper-cell infiltration was also associated with a poor outcome and correlated with CD20+ cell densities. In addition, increased CD45RO+ memory T-cell densities were identified as a positive prognostic indicator. An immunoscore based on CD20+ and CD45RO+ cell densities identified different risk groups. The findings of this study suggest that TIICs may also play a crucial role in the outcome of HR+ BC and highlight the importance of immunological research in this BC subtype.

## Figures and Tables

**Table 1:** Clinical characteristics.

Variables				Groups				
Age (years)	mean: 59.1;			<50: 33 (22.6%);			≥50: 113 (77.4%)	
T category	pT1mic: 10 (6.8%)		pT1a: 10 (6.8%)		pT1b: 38 (26%)		pT1c: 82 (56.2%)	pT2: 6 (4.1%)
N category	N0: 143 (97.9%)			N1: 3 (2.1%)			
Stage	UICC I: 134 (91.8%)			UICC II: 12 (8.2%)				
Tumour size (mm)	<10: 40 (27.4%);			10–20: 96 (65.8%);			>20: 10 (6.8%)	
Histological grading	G1: 38 (26%)		G2: 101 (69.2%)		G3: 4 (2.7%)		n.a. 3 (2.1%)	
Histological typing	lobular: 23 (15.8%)			no special type: 100 (68.5%)			other: 23 (15.8%)	
Ki67	<20: 110 (75.3%)			≥20: 32 (21.9%)			n.a. 4 (2.7%)	
Oestrogen receptor status	positive: 141 (96.6%)			negative: 1 (0.7%)			n.a. 4 (2.7%)	
Progesterone receptor status	positive: 132 (90.4%)			negative: 11 (7.5%)			n.a. 3 (2.1%)	
Her2 status	positive: 8 (5.5%)			negative: 133 (91.1%)			n.a. 5 (3.4%)	
Molecular subtype	Luminal A: 97 (66.4%)			Luminal B: 44 (30.1%)			n.a. 5 (3.4%)	
Hormone therapy	Yes: 131 (89.7%)			No: 15 (10.3%)				
Chemotherapy	Yes: 10 (6.8%)			No: 136 (93.2%)				

n.a. = not available.

**Table 2:** Correlations between stromal CD4+ and CD20+ TIICs in different locations.

		CD20+ TIIC Densities Normal Tissue			CD20+ TIIC Densities	
		Proximity	Distant		Central Tumour	Invasive Front
Correlation coefficient	CD4+ TIIC densities normal tissue (prox)	0.139	0.080	CD4+ TIIC densities central tumour	**0.521**	**0.193**
*p*		0.192	0.459		**<0.001**	**0.038**
n		90	89		**122**	**116**
Correlation coefficient	CD4+ TIIC densities normal tissue (dist)	0.126	0.081	CD4+ TIIC densities invasive front	**0.179**	**0.397**
*p*		0.248	0.373		**0.049**	**<0.001**
n		86	123		**121**	**128**

**Table 3:** Correlations between clinicopathological characteristics and CD45RO+ TIICs in both tumour compartments of invasive front samples.

		Stromal (*n* = 134)			Intraepithelial (*n* = 111)		
	N (total)	CD45RO+ Tiics Low	CD45RO+ TIICs High	*p*	CD45RO+ TIICs Low	CD45RO+ TIICs High	*p*
Age (years)				0.02			0.21
<50	33	1 (4%)	26 (24%)		16 (18%)	6 (30%)	
≥50	113	25 (96%)	82 (76%)		75 (82%)	14 (70%)	
Tumour size (mm)				0.43			0.49
<20	136	25 (96%)	99 (92%)		82 (90%)	19 (95%)	
≥20	10	1 (4%)	9 (8%)		9 (10%)	1 (5%)	
Histological grading				0.57			0.49
G1	38	6 (23%)	30 (29%)		24 (27%)	7 (35%)	
G2 + G3	105	20 (77%)	75 (71%)		64 (73%)	13 (65%)	
n.a.	3						
Histological typing				0.94			0.01
nonlobular	123	22 (85%)	92 (85%)		83 (91%)	14 (70%)	
lobular	23	4 (15%)	16 (15%)		8 (9%)	6 (30%)	
Ki67				0.38			0.43
<20	110	18 (72%)	84 (80%)		67 (77%)	17 (85%)	
≥20	32	7 (28%)	21 (20%)		20 (23%)	3 (15%)	
n.a.	4						
ER status				0.62			0.63
neg	1	0 (0%)	1 (1%)		1 (1%)	0 (0%)	
pos	141	25 (100%)	104 (99%)		86 (99%)	20 (100%)	
n.a.	4						
PR status				0.13			0.47
neg	11	4 (16%)	7 (7%)		9 (10%)	1 (5%)	
pos	132	21 (84%)	99 (93%)		79 (90%)	19 (95%)	
n.a.	3						
Her2 status				0.67			0.94
neg	133	23 (92%)	99 (94%)		83 (95%)	19 (95%)	
pos	8	2 (8%)	6 (6%)		4 (5%)	1 (5%)	
n.a.	5						
Subtype				0.12			0.23
Luminal A	97	14 (56%)	75 (72%)		57 (66%)	16 (80%)	
Luminal B	44	11 (44%)	29 (28%)		29 (34%)	4 (20%)	
n.a.	5						

n.a. = not available.

**Table 4:** Univariate and multivariate analysis of disease-free survival according to Cox’s proportional hazards model.

Breast Cancer	Univariate Analysis			Multivariate Analysis		
Variable	Hazard Ratio	95% C.I.	*p*	Hazard Ratio	95% C.I.	*p*
Age (years) (<50 (*n* = 28) vs ≥50 (*n* = 118))	1.009	0.339–3.002	0.988	---	---	---
Stage (UICC I (*n* = 134) vs UICC II (*n* = 12 ))	1.934	0.569–6.576	0.291	---	---	---
Tumour size (mm) (<20 (*n* = 136) vs ≥20 (*n* = 10))	1.379	0.321–5.927	0.666	---	---	---
Histological grading (G1 (*n* = 38) vs G2-3 (*n* = 105))	3.344	0.779–14.365	0.105	2.216	0.257–19.103	0.469
Histological typing (nonlobular (*n* = 123) vs lobular (*n* = 23))	0.722	0.211–2.471	0.603	---	---	---
DCIS (no (*n* = 76) vs yes (*n* = 57))	0.798	0.312–2.04	0.637	---	---	---
Ki67 (<20 (*n* = 110) vs ≥20 (*n* = 32))	2.563	1.077–6.098	**0.033**	5.973	1.677–21.278	**0.006**
Her2 status (negative (*n* = 133) vs positive (*n* = 8))	0.890	0.119–6.66	0.910	---	---	---
Luminal (A (*n* = 97) vs B (*n* = 44))	2.182	0.925–5.147	0.075	0.273	0.024–3.137	0.298
Hormone therapy (no (*n* = 15) vs yes (*n* = 131))	1.003	0.234–4.309	0.996	---	---	---
Chemotherapy (no (*n* = 136) vs yes (*n* = 10 ))	2.280	0.67–7.763	0.187	---	---	---
Stromal CD4+ TIICs (low (*n* = 59) vs high (*n* = 75))	2.712	0.899–8.185	0.077	1.250	0.154–10.156	0.834
Intraepithelial CD4+ TIICs (low (*n* = 86) vs high (*n* = 25))	5.467	1.963–15.226	**0.001**	2.869	0.929–8.857	0.067
Stromal CD45RO+ TIICs (low (*n* = 26) vs high (*n* = 108))	0.243	0.098–0.599	**0.002**	0.104	0.027–0.398	**0.001**
Intraepithelial CD45RO+ TIICs (low (*n* = 91) vs high (*n* = 20))	0.854	0.191–3.816	0.836	---	---	---
Stromal CD1a+ TIICs (low (*n* = 106) vs high (*n* = 22))	0.545	0.124–2.398	0.422	---	---	---
Intraepithelial CD1a+ TIICs (low (*n* = 58) vs high (*n* = 47))	0.688	0.241 - 1.968	0.486	---	---	---
Stromal CD20+ TIICs (low (*n* = 76) vs high (*n* = 52))	2.535	0.935–6.872	0.067	11.601	2.485–54.152	**0.002**
Intraepithelial CD20+ TIICs (low (*n* = 74) vs high (*n* = 31))	2.132	0.771–5.891	0.144	0.619	0.129–2.969	0.549

## Abbreviations

TIIC	Tumour-infiltrating inflammatory cell
APBI	Accelerated partial breast irradiation
TMA	Tissue microarray
TAM	Tumour-associated macrophage
CT	Central tumour
IF	Invasive front
prox	Normal tissue in tumour proximity
dist	Normal tissue sampled distant from the tumour
HR+/-	Hormone receptor positive/negative
BC	Breast cancer
RT	Radiotherapy
ER	Oestrogen receptor
PR	Progesterone receptor
TNBC	Triple-negative breast cancer
DFS	Disease-free survival
